# Supplementary material for: Convex Hartree–Fock theory for modeling ground state conical intersections
Source: Commun Chem. 2026 Jan 5;9:32. doi: 10.1038/s42004-025-01842-2 (PMC12820363; doi:10.1038/s42004-025-01842-2)
Supplement: Supplementary file 1 — Supplementary Information [file 42004_2025_1842_MOESM1_ESM.pdf]

Supplementary Information for "Convex Hartree-Fock theory for modeling ground state conical intersections"

Federico Rossi[1] and Henrik Koch*[1*]

[1]Department of Chemistry, Norwegian University of Science and Technology, Trondheim, 7491, Norway.

*Corresponding author(s). E-mail(s): henrik.koch@ntnu.no;

Contents

1 Hamiltonian matrix in the new basis **S3**

1.1 Single projected state . . . S3
1.2 Multiple projected states . . . S3

2 Metric matrix **S5**

3 Reduced matrix with additional vectors **S6**

4 Extensivity **S7**

5 Effect of multiple projections **S7**

6 Timings **S9**

7 Geometries **S10**

7.1 Ammonia . . . S10
7.2 2,4-cyclohexadien-1-ylamine . . . S13
7.3 GFP chromophore $HBDI^-$ . . . S16

Supplementary Note 1

Hamiltonian matrix in the new basis

1.1 Single projected state

The full space Hamiltonian matrix with a single projected state is

$$\mathbf{H}^{\mathrm{FS}} = \begin{pmatrix} \langle \mathrm{HF}|H|\mathrm{HF}\rangle & \langle \mathrm{HF}|H|\mathrm{R}_1\rangle & \langle \mathrm{HF}|H|\tilde{\nu}\rangle \\ \langle \mathrm{R}_1|H|\mathrm{HF}\rangle & \langle \mathrm{R}_1|H|\mathrm{R}_1\rangle & \langle \mathrm{R}_1|H|\tilde{\nu}\rangle \\ \langle \tilde{\mu}|H|\mathrm{HF}\rangle & \langle \tilde{\mu}|H|\mathrm{R}_1\rangle & \langle \tilde{\mu}|H|\tilde{\nu}\rangle \end{pmatrix} = \begin{pmatrix} E_0 & \mathbf{F}_{OV}\mathbf{r}_1 & 0 \\ \mathbf{F}_{OV}\mathbf{r}_1 & E_0 + \mathbf{r}_1^T\mathbf{A}\mathbf{r}_1 & V_\nu \\ 0 & V_\mu & Z_{\mu\nu} + \delta_{\mu\nu}E_0 \end{pmatrix} \tag{1}$$

where

$$V_\mu = \langle \tilde{\mu}|H|\mathrm{R}_1\rangle = \langle \mu|H|\mathrm{R}_1\rangle - \langle \mu|\mathrm{R}_1\rangle\langle \mathrm{R}_1|H|\mathrm{R}_1\rangle \tag{2}$$

$$= \sum_\nu (E_0\delta_{\mu\nu} + A_{\mu\nu})r_{1,\nu} - r_{1,\mu}(E_0 + \mathbf{r}_1^T\mathbf{A}\mathbf{r}_1) \tag{3}$$

$$= A_{\mu\nu}r_{1,\nu} - r_{1,\mu}\mathbf{r}_1^T\mathbf{A}\mathbf{r}_1 \tag{4}$$

$$Z_{\mu\nu} = \langle \tilde{\mu}|H|\tilde{\nu}\rangle - \delta_{\mu\nu}E_0 = \langle \mu|H|\nu\rangle - \delta_{\mu\nu}E_0 - \langle \tilde{\mu}|H|\mathrm{R}_1\rangle\langle \mathrm{R}_1|\nu\rangle + \tag{5}$$

$$- \langle \mu|\mathrm{R}_1\rangle\langle \mathrm{R}_1|H|\tilde{\mu}\rangle - \langle \mu|\mathrm{R}_1\rangle\langle \mathrm{R}_1|H|\mathrm{R}_1\rangle\langle \mathrm{R}_1|\nu\rangle \tag{6}$$

$$= A_{\mu\nu} - r_{1,\mu}V_\nu - V_\mu r_{1,\nu} - r_{1,\mu}r_{1,\nu}(E_0 + \mathbf{r}_1^T\mathbf{A}\mathbf{r}_1) \tag{7}$$

1.2 Multiple projected states

The reduced space Hamiltonian matrix with N projected states is defined as

$$\mathbf{H}^{\mathrm{RS}} = \begin{pmatrix} \langle \mathrm{HF}|H|\mathrm{HF}\rangle & \langle \mathrm{HF}|H|\mathrm{R}_1\rangle & \dots & \langle \mathrm{HF}|H|\mathrm{R}_N\rangle \\ \langle \mathrm{R}_1|H|\mathrm{HF}\rangle & \langle \mathrm{R}_1|H|\mathrm{R}_1\rangle & \dots & \langle \mathrm{R}_1|H|\mathrm{R}_N\rangle \\ \vdots & \vdots & \ddots & \vdots \\ \langle \mathrm{R}_N|H|\mathrm{HF}\rangle & \langle \mathrm{R}_N|H|\mathrm{R}_1\rangle & \dots & \langle \mathrm{R}_N|H|\mathrm{R}_N\rangle \end{pmatrix} = \begin{pmatrix} E_0 & \mathbf{F}_{OV}\mathbf{r}_1 & \dots & \mathbf{F}_{OV}\mathbf{r}_N \\ \mathbf{F}_{OV}\mathbf{r}_1 & E_0 + \mathbf{r}_1^T\mathbf{A}\mathbf{r}_1 & \dots & \mathbf{r}_1^T\mathbf{A}\mathbf{r}_N \\ \vdots & \vdots & \ddots & \vdots \\ \mathbf{F}_{OV}\mathbf{r}_N & \mathbf{r}_N^T\mathbf{A}\mathbf{r}_1 & \dots & E_0 + \mathbf{r}_N^T\mathbf{A}\mathbf{r}_N \end{pmatrix} \tag{8}$$

where

$$W_{IJ} = \langle \mathrm{R}_I|H|\mathrm{R}_J\rangle - \delta_{IJ}E_0 = \mathbf{r}_I^T\mathbf{A}\mathbf{r}_J \tag{9}$$

The full space Hamiltonian matrix reads

$$\mathbf{H}^{\mathrm{FS}} = \begin{pmatrix} \langle \mathrm{HF}|H|\mathrm{HF}\rangle & \langle \mathrm{HF}|H|\mathrm{R}_1\rangle & \dots & \langle \mathrm{HF}|H|\mathrm{R}_N\rangle & \langle \mathrm{HF}|H|\tilde{\nu}\rangle \\ \langle \mathrm{R}_1|H|\mathrm{HF}\rangle & \langle \mathrm{R}_1|H|\mathrm{R}_1\rangle & \dots & \langle \mathrm{R}_1|H|\mathrm{R}_N\rangle & \langle \mathrm{R}_1|H|\tilde{\nu}\rangle \\ \vdots & \vdots & \ddots & \vdots & \vdots \\ \langle \mathrm{R}_N|H|\mathrm{HF}\rangle & \langle \mathrm{R}_N|H|\mathrm{R}_1\rangle & \dots & \langle \mathrm{R}_N|H|\mathrm{R}_N\rangle & \langle \mathrm{R}_N|H|\tilde{\nu}\rangle \\ \langle \tilde{\mu}|H|\mathrm{HF}\rangle & \langle \tilde{\mu}|H|\mathrm{R}_1\rangle & \dots & \langle \tilde{\mu}|H|\mathrm{R}_N\rangle & \langle \tilde{\mu}|H|\tilde{\nu}\rangle \end{pmatrix} = \tag{10}$$

$$= \begin{pmatrix} E_0 & \mathbf{F}_{OV}\mathbf{r}_1 & \dots & \mathbf{F}_{OV}\mathbf{r}_N & X_\nu \\ \mathbf{F}_{OV}\mathbf{r}_1 & E_0 + \mathbf{r}_1^T\mathbf{A}\mathbf{r}_1 & \dots & \mathbf{r}_1^T\mathbf{A}\mathbf{r}_N & Y_{1,\nu} \\ \vdots & \vdots & \ddots & \vdots & \vdots \\ \mathbf{F}_{OV}\mathbf{r}_N & \mathbf{r}_N^T\mathbf{A}\mathbf{r}_1 & \dots & E_0 + \mathbf{r}_N^T\mathbf{A}\mathbf{r}_N & Y_{N,\nu} \\ 0 & V_{1,\mu} & \dots & V_{N,\mu} & Z_{\mu\nu} + \delta_{\mu\nu}E_0 \end{pmatrix} \tag{11}$$

where

$$X_\nu = \langle \mathrm{HF}|H|\tilde{\nu}\rangle = \eta_\nu - \sum_I (\boldsymbol{\eta}^T\mathbf{r}_I)\mathrm{R}_{I\nu} \tag{12}$$

$$Y_{I,\nu} = \langle \mathrm{R}_I|H|\tilde{\nu}\rangle = \langle \mathrm{R}_I|H|\nu\rangle - \sum_J (E_0\delta_{IJ} + W_{IJ})\mathrm{R}_{J\nu} \tag{13}$$

$$V_{I,\mu} = \langle \tilde{\mu}|H|\mathrm{R}_I\rangle = \langle \mu|H|\mathrm{R}_I\rangle - \sum_J \mathrm{R}_{J\mu}(E_0\delta_{IJ} + W_{JI}) \tag{14}$$

$$Z_{\mu\nu} = \langle \tilde{\mu}|H|\tilde{\nu}\rangle = \langle \mu|H|\nu\rangle - \delta_{\mu\nu}E_0 - \sum_I \mathrm{R}_{I\mu}Y_{I,\nu} - \sum_I V_{I,\mu}\mathrm{R}_{I\nu} - \sum_{IJ} \mathrm{R}_{I\mu}(E_0\delta_{IJ} + W_{IJ})\mathrm{R}_{J\nu} \tag{15}$$

Supplementary Note 2

Metric matrix

Taking as example the case of a single projected state, we show that the inclusion of the metric matrix is not needed when solving the full space eigenvalue problem

$$\mathbf{H}^{\mathrm{FS}}\mathbf{x}_n = \mathcal{E}_n\mathbf{S}^{\mathrm{FS}}\mathbf{x}_n. \tag{16}$$

We report the matrices defined as

$$\mathbf{H}^{\mathrm{FS}} = \begin{pmatrix} \langle \mathrm{HF}|H|\mathrm{HF}\rangle & \langle \mathrm{HF}|H|\mathrm{R}_1\rangle & 0 \\ \langle \mathrm{R}_1|H|\mathrm{HF}\rangle & \langle \mathrm{R}_1|H|\mathrm{R}_1\rangle & \langle \mathrm{R}_1|H|\tilde{\nu}\rangle \\ 0 & \langle \tilde{\mu}|H|\mathrm{R}_1\rangle & \langle \tilde{\mu}|H|\tilde{\nu}\rangle \end{pmatrix} \tag{17}$$

$$\mathbf{S}^{\mathrm{FS}} = \begin{pmatrix} 1 & \langle \mathrm{HF}|R_1\rangle & \langle \mathrm{HF}|\tilde{\nu}\rangle \\ \langle R_1|\mathrm{HF}\rangle & 1 & \langle R_1|\tilde{\nu}\rangle \\ \langle \tilde{\mu}|\mathrm{HF}\rangle & \langle \tilde{\mu}|R_1\rangle & \langle \tilde{\mu}|\tilde{\nu}\rangle \end{pmatrix} = \begin{pmatrix} 1 & 0 & 0 \\ 0 & 1 & 0 \\ 0 & 0 & \langle \tilde{\mu}|\tilde{\nu}\rangle \end{pmatrix}. \tag{18}$$

In what follows, we will show that the eigenvectors of the standard (non-generalized) eigenvalue problem

$$\mathbf{H}^{\mathrm{FS}}\mathbf{x}_n = \mathcal{E}_n\mathbf{x}_n \tag{19}$$

are such that $\mathbf{S}^{\mathrm{FS}}\mathbf{x}_n = \mathbf{x}_n$, which means that they are also eigenvectors of the generalized eigenvalue problem with the same eigenvalue.

We start by writing explicitly $\mathbf{S}^{\mathrm{FS}}\mathbf{x} - \mathbf{x}$:

$$\mathbf{S}^{\mathrm{FS}}\mathbf{x} - \mathbf{x} = \begin{pmatrix} 1 & 0 & 0 \\ 0 & 1 & 0 \\ 0 & 0 & \langle \tilde{\mu}|\tilde{\nu}\rangle \end{pmatrix} \begin{pmatrix} x_0 \\ x_1 \\ x_\nu \end{pmatrix} - \begin{pmatrix} x_0 \\ x_1 \\ x_\mu \end{pmatrix} = \begin{pmatrix} 0 \\ 0 \\ -r_{1,\mu}r_{1,\nu}x_\nu \end{pmatrix} \tag{20}$$

We are now left to show that $\sum_\mu r_{1,\mu}x_\mu = 0$. Since $\mathbf{x}$ is an eigenvector of $\mathbf{H}^{\mathrm{FS}}$, we know that

$$\langle \tilde{\mu}|\bar{H}|R_1\rangle x_1 + \sum_\nu \langle \tilde{\mu}|\bar{H}|\tilde{\nu}\rangle x_\nu = \mathcal{E}x_\mu \tag{21}$$

and we can isolate x_μ and substitute

$$\sum_\mu r_{1,\mu}x_\mu = \frac{1}{\mathcal{E}}\sum_\mu r_{1,\mu}\langle \tilde{\mu}| \left(\bar{H}|R_1\rangle x_1 + \sum_\nu \bar{H}|\tilde{\nu}\rangle x_\nu \right) \tag{22}$$

$$= \frac{1}{\mathcal{E}}\sum_\mu r_{1,\mu}(\langle \mu| - r_{1,\mu}\langle R_1|) \left(\bar{H}|R_1\rangle x_1 + \sum_\nu \bar{H}|\tilde{\nu}\rangle x_\nu \right) \tag{23}$$

$$= \frac{1}{\mathcal{E}}(\langle R_1| - \langle R_1|) \left(\bar{H}|R_1\rangle x_1 + \sum_\nu \bar{H}|\tilde{\nu}\rangle x_\nu \right) = 0. \tag{24}$$

Note that we assumed $\mathcal{E} \neq 0$ and $\sum_\mu r_{1,\mu}r_{1,\mu} = 1$.

Supplementary Note 3

Reduced matrix with additional vectors

We consider the case of two molecules A and B infinitely separated. The CVX-HF reduced space matrices for the two separated systems are defined as

$$\mathbf{H}_A^{\mathrm{RS}} = \begin{pmatrix} \langle \mathrm{HF}_A|H_A|\mathrm{HF}_A\rangle & \langle \mathrm{HF}_A|H_A|R_A\rangle \\ \langle R_A|H_A|\mathrm{HF}_A\rangle & \langle \mathrm{R}_A|H_A|R_A\rangle \end{pmatrix} = \begin{pmatrix} E_0^A & c_A \\ c_A & E_1^A \end{pmatrix} \tag{25}$$

$$\mathbf{H}_B^{\mathrm{RS}} = \begin{pmatrix} \langle \mathrm{HF}_B|H_B|\mathrm{HF}_B\rangle & \langle \mathrm{HF}_B|H_B|R_B\rangle \\ \langle R_B|H_B|\mathrm{HF}_B\rangle & \langle \mathrm{R}_B|H_B|R_B\rangle \end{pmatrix} = \begin{pmatrix} E_0^B & c_B \\ c_B & E_1^B \end{pmatrix}. \tag{26}$$

For the total system, the reduced matrix extended to include the combined excitation reads

$$\mathbf{H}^{\mathrm{RS}} = \begin{pmatrix} \langle \mathrm{HF}|H|\mathrm{HF}\rangle & \langle \mathrm{HF}|H|R_1\rangle & \langle \mathrm{HF}|H|R_2\rangle & \langle \mathrm{HF}|H|R_1R_2\rangle \\ \langle R_1|H|\mathrm{HF}\rangle & \langle R_1|H|R_1\rangle & \langle R_1|H|R_2\rangle & \langle R_1|H|R_1R_2\rangle \\ \langle R_2|H|\mathrm{HF}\rangle & \langle R_2|H|R_1\rangle & \langle R_2|H|R_2\rangle & \langle R_2|H|R_1R_2\rangle \\ \langle R_1R_2|H|\mathrm{HF}\rangle & \langle R_1R_2|H|R_1\rangle & \langle R_1R_2|H|R_2\rangle & \langle R_1R_2|H|R_1R_2\rangle \end{pmatrix}. \tag{27}$$

Assuming the ordering of the eigenvectors is such that $|R_1\rangle$ is the one located on system A, at infinite distance we have

$$|\mathrm{HF}\rangle = |\mathrm{HF}_A\rangle|\mathrm{HF}_B\rangle \tag{28}$$

$$|R_1\rangle = |R_A\rangle|\mathrm{HF}_B\rangle \tag{29}$$

$$|R_2\rangle = |\mathrm{HF}_A\rangle|R_B\rangle \tag{30}$$

$$|R_1R_2\rangle = |R_A\rangle|R_B\rangle. \tag{31}$$

Using this, the extended reduced matrix for the combined system takes the form

$$\mathbf{H}^{\mathrm{RS}} = \begin{pmatrix} E_0^A + E_0^B & c_A & c_B & 0 \\ c_A & E_1^A + E_0^B & 0 & c_B \\ c_B & 0 & E_0^A + E_1^B & c_A \\ 0 & c_B & c_A & E_1^A + E_1^B \end{pmatrix}. \tag{32}$$

This shows that $\mathbf{H}^{\mathrm{RS}}$ can be expressed as the Kronecker sum

$$\mathbf{H}_B^{\mathrm{RS}} \oplus \mathbf{H}_A^{\mathrm{RS}} = \mathbf{H}_B^{\mathrm{RS}} \otimes \mathbf{I}_2 + \mathbf{I}_2 \otimes \mathbf{H}_A^{\mathrm{RS}}. \tag{33}$$

The eigenvalues of such a Kronecker sum are obtained as the pairwise sum of the eigenvalues of $\mathbf{H}_B^{\mathrm{RS}}$ and $\mathbf{H}_A^{\mathrm{RS}}$. (Theorem 10.1 [1])

Supplementary Note 4

Extensivity

The extensivity of the method is tested numerically on a single molecule of 2,4-cyclohexadien-1-ylamine, adding He atoms at large distances.

n_{He}	E_0	E_1	$E_0 - n_{\mathrm{He}}E_{\mathrm{He}}$	$E_1 - n_{\mathrm{He}}E_{\mathrm{He}}$
0	-286.71831598	-286.64708752	-286.71831598	-286.64708752
1	-289.57347646	-289.50224800	-286.71831598	-286.64708752
2	-292.42863694	-292.35740848	-286.71831598	-286.64708752
3	-295.28379741	-295.21256895	-286.71831598	-286.64708752

Table S1: CVX-HF energy in Hartrees of ground and first excited states of 2,4-cyclohexadien-1-ylamine, adding multiple He atoms separated by 500 a.u. in all directions. On the right, the same energies are reported after removing the HF energy for the corresponding number of isolated He atoms. For a single He atom, $E_{\mathrm{He}} = -2.855160477$ Hartree. The geometry is reported in Table S9. The basis is cc-pVDZ and the converging thresholds are set at 10^{-8} with a single projected state.

Supplementary Note 5

Effect of multiple projections

To study the effect of the inclusion of multiple Hessian eigenvectors in the projection operator, the CVX-HF ground and excited state energies are calculated after progressively increasing the number of projected states n_{proj}. The results for the full matrix energies and excitation energies are reported below.

n_{proj}	E_0	E_1	E_2	E_3	E_4	E_5
TDA-TDHF	-719.277718	-719.277431	-719.237085	-719.127870	-719.115821	-719.110472
1	-719.277870	-719.277718	-	-	-	-
2	-719.277725	-719.277717	-719.236711	-	-	-
3	-719.277313	-719.276764	-719.236363	-719.128231	-	-
4	-719.275613	-719.274790	-719.234622	-719.127736	-719.114103	-
5	-719.275568	-719.274900	-719.234536	-719.128065	-719.114284	-719.108815

Table S2: CVX-HF energy in Hartrees of ground and excited states of $HBDI^-$ when increasing the number of vectors included in the projection operator. The molecule is close to the conical intersection and the geometry is reported in Table S14. TDA-TDHF energies are also shown in the first row. The basis is 6-31G* and the converging thresholds are set at 10^{-6}.

n_{proj}	ω_1	ω_2	ω_3	ω_4	ω_5
TDA-TDHF	0.007817	1.105695	4.077571	4.405435	4.551006
1	0.004151	-	-	-	-
2	0.000212	1.116030	-	-	-
3	0.014954	1.114308	4.056745	-	-
4	0.022385	1.115429	4.023947	4.394918	-
5	0.018170	1.116529	4.013757	4.388774	4.537569

Table S3: CVX-HF excitation energies in eV for $HBDI^-$ when increasing the number of vectors included in the projection operator. The molecule is close to the conical intersection and the geometry is reported in Table S14. TDA-TDHF excitation energies are also shown in the first row. The basis is 6-31G* and the converging thresholds are set at 10^{-6}.

n_{proj}	E_0	E_1	E_2	E_3	E_4	E_5
TDA-TDHF	-286.875667	-286.681757	-286.592362	-286.574532	-286.569805	-286.567787
1	-286.875662	-286.681755	-	-	-	-
2	-286.875661	-286.681760	-286.592332	-	-	-
3	-286.874821	-286.679887	-286.591969	-286.574222	-	-
4	-286.874807	-286.679857	-286.591975	-286.574195	-286.569511	-
5	-286.874803	-286.679873	-286.591930	-286.574137	-286.569497	-286.568915

Table S4: CVX-HF energy in Hartrees of ground and excited states of 2,4-cyclohexadien-1-ylamine when increasing the number of vectors included in the projection operator. The molecule is in the Franck-Condon region and the geometry is reported in Table S13. TDA-TDHF energies are also shown in the first row. The basis is cc-pVDZ and the converging thresholds are set at 10^{-6}.

n_{proj}	ω_1	ω_2	ω_3	ω_4	ω_5
TDA-TDHF	5.276549	7.709129	8.194292	8.322940	8.377833
1	5.276503	-	-	-	-
2	5.276322	7.709766	-	-	-
3	5.304423	7.696792	8.179718	-	-
4	5.304865	7.696271	8.180077	8.307540	-
5	5.304309	7.697371	8.181555	8.307805	8.323645

Table S5: CVX-HF excitation energies in eV for 2,4-cyclohexadien-1-ylamine when increasing the number of vectors included in the projection operator. The molecule is in the Franck-Condon region and the geometry is reported in Table S13. TDA-TDHF excitation energies are also shown in the first row. The basis is cc-pVDZ and the converging thresholds are set at 10^{-6}.

Supplementary Note 6

Timings

We report the number of iterations and timings when comparing TDA-TDHF and CVX-HF in a system close to a conical intersection (Table S6) and one in the Franck-Condon region (Table S7). For the ground state, we provide the number of iterations of the Newton-Raphson solver. In the case of CVX-HF, the reported time includes the determination of the lowest Hessian eigenvector performed at every iteration. For the excited state, the number of iterations of the Davidson solver are reported for both methods. The timings are wall times in seconds for the entire calculation on an Intel Xeon Gold 6342 using 24 cores.

Table S6: Information about convergence for $HBDI^-$ close to a conical intersection. The geometry is the same as Tables S2-S3 and reported in Table S14. For both methods, 6-31G* basis was used, solving for one excited state with thresholds of 10^{-6}.

Method	Energy	Iterations	Time	Total time
TDA-TDHF	Ground state	12	149.4 s	165.9 s
	Excited state	13	16.0 s	
CVX-HF	Ground state	8	305.9 s	355.8 s
	Excited state	12	49.5 s	

Table S7: Information about convergence for 2,4-cyclohexadien-1-ylamine in the Franck-Condon region. The geometry is the same as Tables S4-S5 and reported in Table S13. For both methods, cc-pVDZ basis was used, solving for one excited state with thresholds of 10^{-6}.

Method	Energy	Iterations	Time	Total time
TDA-TDHF	Ground state	4	10.9 s	16.0 s
	Excited state	13	4.8 s	
CVX-HF	Ground state	5	41.4 s	54.0 s
	Excited state	12	12.3 s	

For the ground state calculation, the total time is increased due to the additional determination of the lowest Hessian eigenvector at every iteration. The overall scaling is the same, but with a prefactor of 3.8 when the molecule in the Franck-Condon region. On the other hand, when we are close to a conical intersection, the additional cost is partially balanced with an improvement in convergence, which leads to less iterations and a scaling prefactor of 2.0. For the excited state problem, we once again have the same overall scaling but different prefactors, 3.1 when near the conical intersection and 2.6 in the Franck-Condon region. The main reason is that for CVX-HF we have to solve for one additional state, to determine the contribution of projected component to the ground state.

Supplementary Note 7

Geometries

All 2D scans are run with the initial geometry, **g** and **h** vectors reported in the following. These vectors are originally determined in Hartree/Bohr and later used as displacement vectors in Bohr, defining a new geometry $\mathbf{r}_0 + \alpha\mathbf{g} + \beta\mathbf{h}$ from the initial geometry $\mathbf{r}_0$.
Conversion factors: 1Å = 1.8897259886 Bohr, 1 Hartree = 27.2114079527 eV

7.1 Ammonia

Table S8: Ammonia CVX-HF/aug-cc-pVDZ S_0/S_1 conical intersection geometry in Angstrom, corresponding to $r_1 = 2.375131$ Å and $\alpha = 90°$.

Atom	x	y	z
N	0.00000000	0.00000000	0.00000000
H	2.37513100	0.00000000	-0.00000000
H	-0.52000000	0.90066642	-0.00000000
H	-0.52000000	-0.90066642	-0.00000000

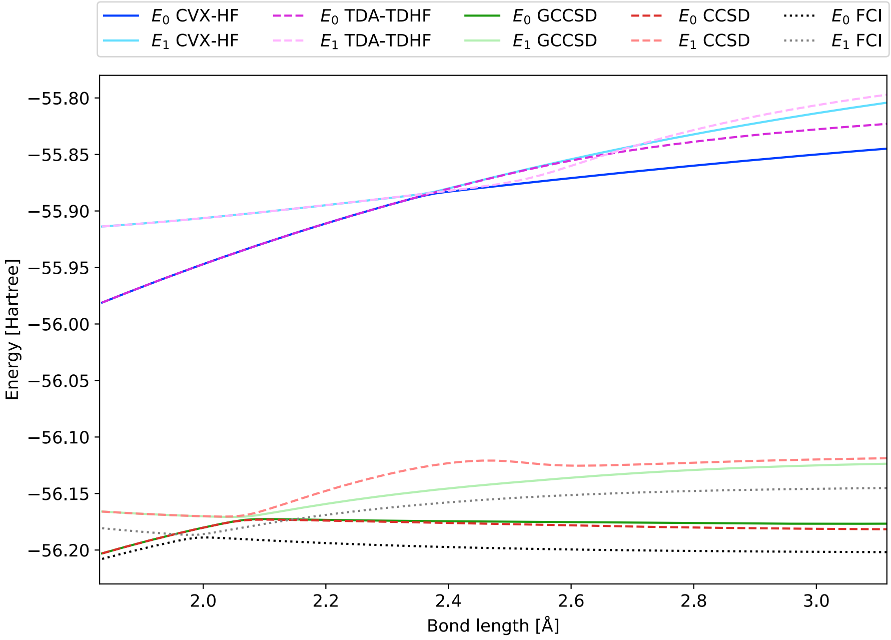

Fig. S1: Potential energy curves of S_0 and S_1 in NH_3 using 6-31G* for different methods. One N-H bond length is stretched with a constant out-of-plane angle $\alpha = 89.5^\circ$ (see Fig. 1 for more information). All energies are expressed in Hartrees.

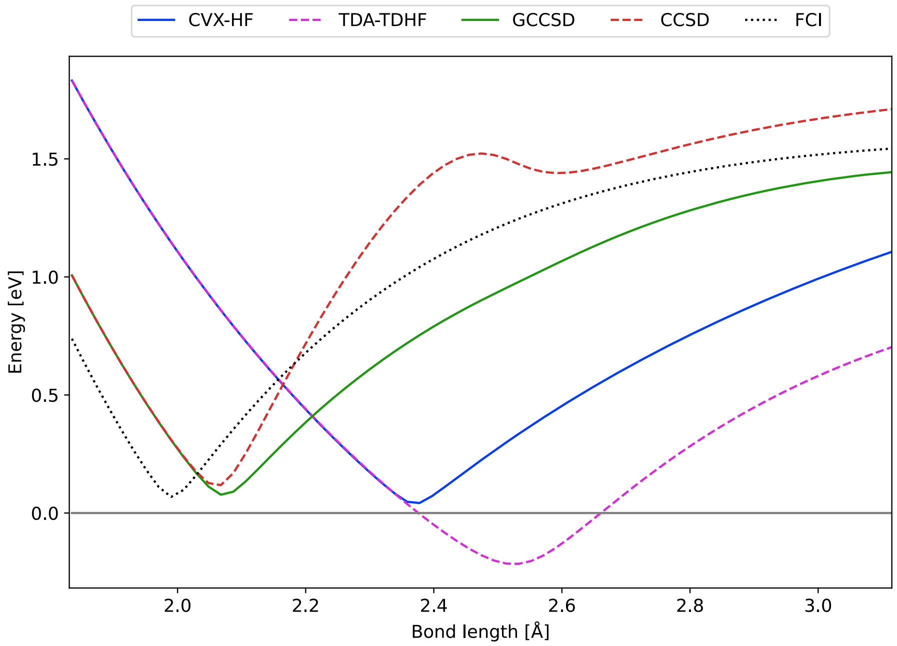

Fig. S2: Excitation energy for the S_0-S_1 transition in NH_3 using 6-31G* for different methods. One N-H bond length is stretched with a constant out-of-plane angle $\alpha = 89.5°$ (see Fig. 1 for more information). All energies are expressed in eV.

7.2 2,4-cyclohexadien-1-ylamine

The initial structure is obtained from Ref. 2 from which the **g** and **h** vectors are calculated at CCSD/6-31G level [3]. Additional details can be found in Ref. 4.

Table S9: 2,4-Cyclohexadien-1-ylamine initial geometry in Bohr.

Atom	x	y	z
N	2.485229105603	0.547832318017	-1.016626362773
C	-1.020180309090	-2.514973398583	-1.130137584081
C	0.000000000000	0.000000000000	0.000000000000
C	-0.152827830887	-4.842609039125	0.243271218683
C	0.000000000000	0.000000000000	2.948948714717
C	0.393673950618	-4.812906418385	2.772429176890
C	0.638744772120	-2.397590397164	4.027981665337
H	3.068814728580	2.219087393160	-0.340105507921
H	3.743103862610	-0.735412398662	-0.407797247778
H	-0.597826785001	-2.656733416686	-3.131827502962
H	-1.241628965952	1.485927944692	-0.652347722798
H	-3.073407175968	-2.431583674518	-0.951269929831
H	-0.051896980885	-6.598968101875	-0.785039323552
H	-2.020803718174	-1.042080601584	3.045064771778
H	0.977265443085	-6.512166056261	3.733568986966
H	1.430957011893	-2.465032088650	5.918242701070

Table S10: 2,4-Cyclohexadien-1-ylamine **g** vector in Hartree/Bohr.

Atom	x	y	z
N	0.004971062452	-0.000006312063	-0.008307843382
C	-0.016216875814	-0.011028497968	0.013811359333
C	0.028843656521	0.052451171540	0.008092929065
C	-0.002450100320	-0.007281793260	-0.010822870907
C	-0.081339063409	-0.074869115270	-0.049735972788
C	-0.013448003251	0.032461991297	0.014876665117
C	0.068149686093	-0.043067069541	0.067549127712
H	0.000405335195	-0.001041323709	-0.002325561626
H	-0.001324788614	-0.000710206799	-0.000031584236
H	0.003448251125	0.006602895615	0.002116067133
H	-0.001001097065	-0.000044020470	-0.002328844295
H	0.003064563939	0.003521849557	-0.003645421539
H	0.001814047671	0.001543968627	-0.001141347406
H	-0.009685201838	0.038125491323	-0.021492693576
H	-0.000178824202	0.003941613764	0.002797133980
H	0.014947351518	-0.000600642643	-0.009411142586

Table S11: 2,4-Cyclohexadien-1-ylamine **h** vector in Hartree/Bohr.

Atom	x	y	z
N	0.002487591355	0.001059423611	-0.001229774795
C	0.001417281298	0.007961593644	0.001946831613
C	0.002788554845	-0.012082019030	-0.001934955082
C	0.000037767487	-0.003593455041	0.002688939505
C	-0.010315266883	-0.006727478406	-0.008749189841
C	0.018041726684	0.000359400262	-0.009740084068
C	0.007161006545	-0.003086550689	-0.006418446171
H	0.000523768377	0.000163090505	0.000341198029
H	0.000201834734	0.000043417177	-0.000047450886
H	0.000287627165	0.001485980656	0.000635812663
H	0.000112190184	0.000739350784	0.000996160267
H	-0.001339158720	-0.002603931746	-0.001285345959
H	-0.003490679658	-0.000662035975	0.000729035443
H	-0.004741496715	0.016752015594	0.017562156627
H	-0.001039863972	-0.000167499259	0.000074916208
H	-0.012149841791	0.000529111955	0.004488870984

Table S12: 2,4-Cyclohexadien-1-ylamine S_0/S_1 CI for CVX-HF/cc-pVDZ in Bohr. The geometry corresponds to $(g, h) = (2.2662, 2.7257)$.

Atom	x	y	z
N	2.503274956386	0.550705684763	-1.038805596200
C	-1.053067911792	-2.518265265119	-1.093531800000
C	0.072966263599	0.085932891857	0.013066089720
C	-0.158277305785	-4.868905721308	0.226073669805
C	-0.212446923726	-0.188005490443	2.812389376610
C	0.412374421419	-4.738361431050	2.779594128750
C	0.812704358800	-2.503602008999	4.163566749585
H	3.071160934833	2.217172081022	-0.344445692522
H	3.740651767411	-0.736903527218	-0.407998160868
H	-0.589228372319	-2.637719595801	-3.125299036580
H	-1.243591855477	1.487843434072	-0.654910115883
H	-3.070112405855	-2.430699995748	-0.963034652450
H	-0.057300531986	-6.597273671507	-0.785638713180
H	-2.055676222684	-0.910019634741	3.044227199854
H	0.974025834216	-6.503690123269	3.740112051570
H	1.431713876188	-2.464951064546	5.909150484729

Table S13: 2,4-Cyclohexadien-1-ylamine S_0 minimum geometry in Bohr as reported in Ref. 2.

Atom	x	y	z
N	5.746192272176	-4.620975638291	-0.583424595068
C	1.222169032706	0.638143504731	-0.206346754446
C	-1.153785513436	-0.692629978080	0.284309295441
C	3.445494179219	-0.636087482707	-0.206393997599
C	-1.251123416386	-3.251372063073	0.152054922887
C	3.520656146097	-3.406119845687	0.474916520995
C	1.074263948388	-4.733042066656	-0.575238301496
H	1.177894639333	2.641275873483	-0.572711737667
H	-2.829553066726	0.388593831625	0.698987016763
H	5.201628005134	0.318743884883	-0.600330084978
H	-3.002132305052	-4.240717388601	0.470061814581
H	0.997682797190	-6.654596848337	0.140688220248
H	3.426449519335	-3.560538815956	2.534848387874
H	1.263329157425	-4.820906772544	-2.620156294315
H	5.772752372856	-6.460830000702	-0.130842747139
H	7.324504759495	-3.861417230057	0.140640977095

7.3 GFP chromophore HBDI$^-$

The initial structure is obtained starting from the P90 MECI in Ref. 5 but rotating ϕ_P by -0.2143° and ϕ_I by -85.8572°. ϕ_P was rotated from the initial 72.6539° to 72.4396° and ϕ_I was rotated from the initial 17.0042° to -68.8530°. Only the dihedral angles have been modified, whereas the structure of the 2 rings and the methine bridge have been kept rigid.

Table S14: HBDI$^-$ initial rotated geometry in Bohr, corresponding to $(\phi_P, \phi_I) = (72.4396°, -68.8530°)$.

Atom	x	y	z
O	7.776918745291	2.323611600006	9.204555374979
O	-0.316144826765	-4.461287451643	-1.205721068545
N	0.084272957587	-1.095075728299	-4.003423314879
N	0.293487044206	2.161259791095	-1.503125191308
C	6.234066932027	1.763049281526	7.586684880001
C	5.279207445979	-0.800572421409	7.342917900429
C	5.236366469809	3.650534445580	5.849145476319
C	3.507734462905	-1.344098324294	5.635763196084
C	3.386703158239	3.058477009156	4.236352804112
C	2.348485142698	0.560089439764	4.111090219886
C	-0.000000000000	-0.000000000000	2.702372449512
C	0.000000000000	0.000000000000	0.000000000000
C	-0.123310731504	-2.078439309446	-1.561220337633
C	0.354697678536	1.410410139114	-3.849773724303
C	0.196612068388	-2.600436588752	-6.255108094215
C	0.702012236290	3.014049280019	-6.103611154541
H	-1.311084192149	-2.100296981874	-7.537904373783
H	2.211096465899	2.294755213707	-7.281723054175
H	1.175547633126	4.901824539753	-5.516261203737
H	6.012296020762	-2.188866080853	8.617178908818
H	6.044064180588	5.500185984979	5.968132582479
H	2.800290110860	-3.217494907645	5.448520092057
H	2.593532959849	4.430645443254	2.985870739078
H	-1.572794122697	0.983887956163	3.526046208710
H	1.992918293691	-2.350373263371	-7.197474982867
H	0.008250794015	-4.545316712444	-5.697112954956
H	-1.000098406522	3.075961326636	-7.239007006443

Supplementary References

[1] Lyche, T. *Numerical linear algebra and matrix factorizations* Vol. 22 (Springer Nature, 2020).

[2] MacDonell, R. J. Polyene meci dataset (2019). URL https://github.com/ryjmacdonell/polyene-meci-dataset.git. Date of access: 2024-12-09.

[3] Angelico, S., Kjønstad, E. F. & Koch, H. Determining minimum energy conical intersections by enveloping the seam: exploring ground and excited state intersections in coupled cluster theory. *J. Phys. Chem. Lett.* **16**, 561–567 (2025).

[4] Rossi, F., Kjønstad, E. F., Angelico, S. & Koch, H. Generalized coupled cluster theory for ground and excited state intersections. *J. Phys. Chem. Lett.* **16**, 568–578 (2025).

[5] Jones, C. M., List, N. H. & Martínez, T. J. Resolving the ultrafast dynamics of the anionic green fluorescent protein chromophore in water. *Chem. Sci.* **12**, 11347–11363 (2021).
